# The contribution of *cis-* and *trans-*acting variants to gene regulation in wild and domesticated barley under cold stress and control conditions

**DOI:** 10.1093/jxb/eraa036

**Published:** 2020-01-28

**Authors:** Matthew Haas, Axel Himmelbach, Martin Mascher

**Affiliations:** 1 Leibniz Institute of Plant Genetics and Crop Plant Research (IPK) Gatersleben, Corrensstraße 3, D-06466 Seeland, Germany; 2 German Center for Integrative Biodiversity Research (iDiv) Halle-Jena-Leipzig, D-04103 Leipzig, Germany; 3 CSIRO Agriculture and Food, Australia

**Keywords:** Allele-specific expression, barley, cold stress, domestication, gene regulation, *Hordeum vulgare*

## Abstract

Barley, like other crops, has experienced a series of genetic changes that have impacted its architecture and growth habit to suit the needs of humans, termed the domestication syndrome. Domestication also resulted in a concomitant bottleneck that reduced sequence diversity in genes and regulatory regions. Little is known about regulatory changes resulting from domestication in barley. We used RNA sequencing to examine allele-specific expression in hybrids between wild and domesticated barley. Our results show that most genes have conserved regulation. In contrast to studies of allele-specific expression in interspecific hybrids, we find almost a complete absence of *trans* effects. We also find that *cis* regulation is largely stable in response to short-term cold stress. Our study has practical implications for crop improvement using wild relatives. Genes regulated in *cis* are more likely to be expressed in a new genetic background at the same level as in their native background.

## Teaser

We present evidence for a near absence of *trans* effects controlling gene regulation in response to domestication and environmental stress

## Introduction

Barley (*Hordeum vulgare* ssp. *vulgare* L.) is an important crop for feed, malting, and, to a lesser extent, human consumption (Ullrich, 2010). Among the first crops to be domesticated in the Fertile Crescent ~10 000 years ago (Zohary *et al.*, 2012), barley remains fully interfertile with its wild progenitor *H. vulgare* ssp. *spontaneum* K. Koch (*H. spontaneum* for short). Therefore, *H. spontaneum* is considered to be a useful source of beneficial alleles for barley improvement. Preferential selection of genotypes with traits beneficial to humans and intentional breeding have narrowed the genetic diversity and altered gene expression patterns. These molecular changes have caused differences in plant architecture and growth habit between wild and domesticated relatives, collectively called the domestication syndrome (Hammer, 1984; Doebley *et al.*, 2006).

In barley, key domestication and crop evolution genes include *Non-brittle rachis 1* (*btr1*) and *Non-brittle rachis 2* (*btr2*) controlling dehiscence of spikelets from the rachis; *six-rowed spike 1* (*vrs1*), which is responsible for lateral floret fertility and may be modified by *INTERMEDIUM-C* (*INT-C*); *VERNALIZATION1* (*Vrn1*) which controls the vernalization requirement; covered/naked caryopsis (*nud*) affecting the adherence of the hull to the caryopsis; and *Photoperiod-H1* (*Ppd-H1*) affecting photoperiod sensitivity (Trevaskis *et al.*, 2003; Turner *et al.*, 2005; Komatsuda *et al.*, 2007; Taketa *et al.*, 2008; Ramsay *et al.*, 2011; Pourkheirandish *et al.*, 2015). These genes were cloned using traditional mapping approaches as their effects are easy to observe given the major phenotypic effect of each gene; however, these tasks were also facilitated by the relative ease with which DNA sequence variation is detected between unrelated genotypes. The task of detecting regulatory variation is more challenging since DNA sequence data alone cannot be used to predict expression. Regulatory variation may arise due to differences in *cis* or *trans* factors. *Cis* factors are physically linked to the genes they control, such as promoters or enhancers, while *trans* factors act distally, such as transcription factors (TFs). Many studies have been conducted to study the effect of domestication on gene regulation (Rapp *et al.*, 2010; Swanson-Wagner *et al.*, 2012; Koenig *et al.*, 2013), although these studies were not designed to disentangle *cis* and *trans* effects.

In order to achieve separation of *cis-* and *trans-*acting factors, Cowles *et al.* (2002) proposed the comparison of allele-specific expression (ASE) in F_1_ hybrids with that of the parents. Subsequently, Wittkopp *et al.* (2004) demonstrated how to find the relative contribution of *cis* and *trans* factors. We show this in Supplementary Fig. S1 at *JXB* online and provide further explanation in the Materials and methods. Zhang and Borevitz (2009) conducted a similar study using a custom gene expression array with allele-specific probes; however, arrays are known to suffer from ascertainment bias (Nielsen, 2000). In addition, it can be challenging to design suitable probes that can distinguish between two alleles as demonstrated in yeast by Tirosh *et al.* (2009). The advent of low-cost RNA sequencing (RNA-seq) enabled the strategy of genome-wide total expression and ASE to be implemented in a single experiment in *Drosophila* (McManus *et al.*, 2010). Lemmon *et al.* (2014) extended this approach to examine regulatory changes between maize and its wild progenitor, teosinte. Cubillos *et al.* (2014) used the approach to examine the steady-state stress drought response in Arabidopsis. To the best of our knowledge, only one previous study has been published examining ASE in barley (von Korff *et al.*, 2009). In that study, the authors used custom gene expression arrays to measure ASE ratios for 30 stress response genes in five F_1_ hybrids at different developmental stages. In the present study, we used RNA-seq to estimate the impact of domestication on gene regulation in barley and whether the response to an environmental stress (cold) is affected by domestication.

## Materials and methods

### Growth conditions

**Fig. 1.**
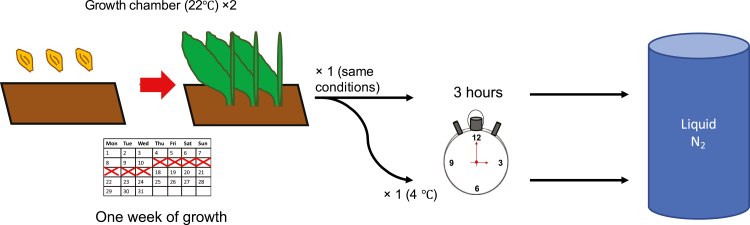
The experimental design. Barley seedlings were grown for 1 week (represented by the calendar) until the first leaf was fully expanded in duplicated trays. The photoperiod in the growth chamber was set to 12 h to avoid the long-day photoperiod response, which could complicate our results. After 1 week of growth, one tray was moved to a cold room (4 °C) for 3 h (from 11.00 h to14.00 h) while the other tray remained in the growth chamber (22 °C). The cold treatment and harvest were conducted at the same time to avoid possible confounding effects of circadian rhythm. After 3 h, samples were collected in liquid nitrogen and stored at –80 °C until we prepared them for RNA extraction. Experiments were conducted during the summer of 2016.

Plants were grown in a growth chamber with a 12 h photoperiod with temperatures of 22 °C and 18 °C during light and dark periods, respectively. After 1 week of growth, when the first leaf of each accession was fully expanded, half of the plants were moved to a cold room at 4 °C for 3 h. The response to chilling occurs rapidly in barley (Cattivelli and Bartels, 1989), so this short cold treatment is sufficient to induce a physiological response. After the 3 h cold treatment, the first leaf of each individual from both groups was harvested and immediately frozen in liquid nitrogen before being moved to storage at –80 °C. Each cold treatment (11.00 h) and tissue harvest (14.00 h) was conducted at the same time of the day for each replicate to avoid confounding factors associated with circadian rhythm. The experimental design is shown in Fig. 1. The experiment was replicated four times. For accessions that either failed to germinate or grew poorly, a fifth attempt was made to obtain additional replicates. As a result, most samples were replicated four times. A few samples have only three replicates: FT67 hybrid cold, FT581 parent control, both FT581 hybrid control and cold, and Morex parent control. Two samples have only two replicates: Barke hybrid control and Igri hybrid cold. One sample, Barke hybrid cold, was not able to be replicated despite repeated efforts to obtain more data.

### RNA extraction, sequencing, and data analysis

Frozen leaf tissue (–80 °C) was homogenized by grinding to a fine powder in 1.5 ml tubes with metal beads twice for 30 s each (1 min total) at 30 Hz using a mixer mill (Retsch GmbH, Haan, Germany). Tubes containing the samples were submerged in liquid nitrogen between grinding to ensure that samples did not thaw during the process. Once all samples were ground, RNA was extracted using RNeasy^®^ mini kits (Qiagen) according to the manufacturer’s instructions. To remove any DNA contamination, samples were treated with Ambion™ DNase (ThermoFisher Scientific) according to the manufacturer’s instructions. RNA quality and integrity were checked with an Agilent 2100 Bioanalyzer (Agilent Technologies) and a Qubit™ 2.0 fluorometer (ThermoFisher Scientific), respectively.

Where possible, three individuals of each parent or hybrid were planted for each replicated treatment. The healthiest plant (e.g. not yellow or stunted) was selected for harvesting. After RNA extraction was carried out according to the methods described above; high quality RNA [mass ≥1 μg, volume ≥20 μl, concentration ≥50 ng μl^–1^, RNA integrity number (RIN) ≥6.3, and 260/280 and 260/230 ≥2.0] samples were submitted for sequencing.

In total, 123 NEB Next^®^ Ultra™ RNA libraries with an average insert size of 250–300 bp were sequenced (paired-end, 2× 150 cycles) on an Illumina HiSeq 2500 machine. RNA sequencing was done by Novogene while exome capture sequencing was performed at the IPK sequencing center. RNA-seq data were quantified using both the pseudoalignment software kallisto v. 0.43.0 (Bray *et al.*, 2016) and HISAT2 (Kim *et al.*, 2015). The abundance files from kallisto and HISAT2 were separately loaded into the R statistical environment (R Core Team, 2012) for further analysis. Gene abundance estimates from kallisto were normalized using edgeR and limma (Robinson *et al.*, 2010; Ritchie *et al.*, 2015), and the voom transformation (Law *et al.*, 2014) was applied to account for the mean–variance relationship of RNA-seq data. These data were used to calculate the variance using the matrixStats package (Bengtsson, 2016). The 1000 genes with the highest variance were used for principal coordinate analysis (PCA). Kallisto was used to find overall expression patterns while HISAT2 was used for allele-specific expression. All raw RNA-seq data are available from the European Nucleotide Archive (ENA) under accession number PRJEB29972. Accession numbers for individual samples are provided in Supplementary Table S1.

### DNA extraction and exome capture

In order to select high-confidence variants for allele-specific expression analysis using a genomic control, an exome capture assay was applied for the eight hybrid genotypes (Mascher *et al.*, 2013). Exome capture data for the parental genotypes can be found in Russell *et al.* (2016). The raw sequence data for these parents were deposited in the ENA and the accession codes are available in Supplementary table 1 of Russell *et al.* (2016). For the present study, hybrid DNA was extracted using a DNeasy^®^ kit (Qiagen). DNA concentrations were measured using a Qubit™ 2.0 fluorometer (ThermoFisher Scientific) and all samples were >20 ng μl^–1^. DNA integrity was verified using a 0.7% agarose gel, which showed that DNA from each sample was intact. Sequencing was performed using an Illumina Hiseq 2500 machine (2× 100 bp, insert size=320 bp). Captured reads were mapped against the bacterial artificial chromosome (BAC)-based Morex reference sequence (Mascher *et al.*, 2017) with BWA-MEM (Li, 2013, Preprint). Coverage was determined by the depth command from SAMtools (Li, 2011) using only properly paired reads. Mapping statistics are available in Supplementary Fig. S3. Raw DNA sequence data are available through the ENA under accession number PRJEB29973.

### Allele-specific transcript quantification and normalization

The R package limma (Ritchie *et al.*, 2015) was used for the analysis of ASE using a linear model approach. Briefly, allele-specific counts were converted into a matrix and rounded to the nearest integer. Counts were then normalized using edgeR (Robinson *et al.*, 2010) to account for differences in total read count between samples, and stored in a differential expression list. A design matrix was created using each combination of generation×accession×treatment as a single factor. The voom transformation was applied to the count matrix to account for the mean–variance relationship of RNA-seq data. The linear model was created by fitting the voom-transformed (Law *et al.*, 2014) count matrix to the design matrix. Differential expression between alleles was identified using the contrasts specified in the contrast matrix. For example, the expression level of each individual parent was contrasted to Morex to decide whether the parents were different from each other. Subsequently, the parental alleles within the hybrid were compared with each other to decide if their expression was different.

### Variant calling and assignment of regulatory categories

To find variants between samples, SNPs were called from sorted and indexed binary alignment map (BAM) files originating from exome capture and RNA-seq samples. The BAM files were sorted and indexed using Novosort (http://www.novocraft.com/products/novosort). A joint single nucleotide polymorphism (SNP) calling across exome capture and RNA-seq was done with SAMtools. The resultant VCF file was imported into R for further analysis.

Allele-specific counts were derived from SNPs in the RNA-seq data that were corroborated by a genomic control. First, informative SNPs were detected in the exome capture data of the parents and one hybrid individual. SNPs were considered informative in a specific cross if (i) the parents carried different alleles in a homozygous state supported by at least two reads and (ii) the hybrid had a heterozygous genotype call that was supported by at least six reads. Genotype calls were made based on allelic ratios extracted from DV (depth of variant allele)/DP (total depth) fields in the VCF. If the variant allele was supported by >20% (>80%) of the reads, the genotypes was called homozygous for the reference (alternative) allele. If the variant allele was present in 30–70% of the reads, a heterozygous genotype was called. We did not impose thresholds on missing rates per site as successful genotype calls were required only for three samples relevant to a single cross (Morex, other parent, F_1_ hybrid).

After ascertaining high-confidence SNPs in the exome capture data, we determined how many reads supported the reference allele or the alternative allele in RNA-seq data for parents and hybrids, and calculated the DV/DP ratio. Information for multiple SNPs was combined at the gene level by merging the SNP information with gene information in the R statistical environment and summing up DP and DV values for all SNPs in a gene. Low DV/DP ratios indicate that more reads originated from the reference (maternal=Morex) allele, while a high DV/DP ratio indicates that more reads originated from the alternative (paternal) allele. A DV/DP ratio of 0.5 means that both alleles are expressed equally. Genes with <50 reads across all samples were filtered out before further analysis. Tables with informative markers and allele-specific read counts in RNA-seq data are available under Digital Object Identifier (DOI) https://doi.org/10.5447/ipk/2020/1 and registered with e!DAL (Arend *et al.*, 2014).

To find genes showing allelic imbalances, a design matrix was created by considering each combination of accession, generation, and treatment as a single factor. The linear model was created by fitting the model specified in the design matrix to the voom-transformed (Law *et al.*, 2014) count matrix. Genes may be assigned to one of seven regulatory categories described by McManus *et al.* (2010). Genes with significant [false discovery rate (FDR) adjusted *P*-value ≤0.01 using the Benjamini–Hochberg procedure] expression differences between parents and parental allele expression levels matching that of their respective parent in the hybrid were assigned to the *cis* only category (see Supplementary Fig. S1A). In contrast, genes with significant expression differences between parents, but not between parental alleles in the hybrid, were assigned to the *trans* only category (Supplementary Fig. S1B). Supplementary Fig. S1C and D shows the expectations for *cis*+*trans* and *cis*×*trans* categories, respectively. Full descriptions of regulatory categories may be found in McManus *et al.* (2010).

### Dominant versus additive inheritance

We used our gene expression data set to find whether genes were inherited in a dominant or an additive manner. We use the classifications given by Albert *et al.* (2018) to make assignments. First, we used the subset of differentially expressed genes (using overall, not allele-specific, expression in parent and hybrids) from each cross as described above. Genes were assigned as Morex dominant if the expression of the gene in the hybrid was greater than in the low parent and matching the expression of Morex. Genes were called recessive when the expression in the hybrid was lower than Morex and matched that of the low parent. We renamed these as ‘paternal allele dominant’ in the final tables. Additive genes were those genes which had intermediate expression values between the two parental alleles. Genes which had higher expression values than both parents and Morex was the high parent were placed into the Morex overdominant category. Genes which had higher expression values than both parents and the paternal parent was the high parent were assigned to the ‘paternal allele overdominant’ category. For the genes which remained unclassified, we used log_2_ fold change (FC) expression values below 1 and greater than –1 for each contrast to assign these genes to the ‘ambiguous’ classification. Even after this step, some genes remained unassigned. We report these genes as ‘not assigned’. The number of genes in each category was small, but for two crosses (Barke and FT67) the number of unassigned genes was relatively high at 174 and 101, respectively.

## Results

### Experimental design

The experimental design is summarized in Fig. 1. Plants were grown in duplicated trays for 1 week in a growth chamber (22 °C day/18 °C night) with a 12 h photoperiod. On the day of the cold treatment, one of the trays was moved to a vernalization chamber (4 °C) for 3 h (11.00–14.00 h). The cold treatment and tissue harvesting were done at the same time of each day to avoid confounding factors due to circadian rhythm. The experiment was conducted four times. A fifth replicate was added in order to get additional replicates for samples which failed during the previous four attempts. For randomization, the layout of plants in the trays was changed for each replicate, but both trays within a replicate had identical layouts.

### Plant material

Three cultivars, two landraces, and four wild accessions were used in this study for a total of nine parental lines (Table 1). This includes the common maternal reference, Morex (CIho 15773), a six-rowed spring malting cultivar from Minnesota, USA (Rasmusson and Wilcoxson, 1979). Each parental genotype was previously subjected to at least two rounds of single-seed descent to decrease residual heterozygosity (Russell *et al.*, 2016). All other accessions were crossed to Morex, bringing the total number of genotypes to 17. Morex was selected because the recently released barley reference genome was generated from BAC sequences originating from this cultivar (Mascher *et al.*, 2017). The other accessions were selected from an exome capture panel of 267 wild and domesticated barleys in order to maximize geographic and genetic diversity (Russell *et al.*, 2016). The target space is 60 Mb or ~75% of the barley gene space (Mascher *et al.*, 2013).

**Table 1.** Accessions used in this research

Accession	Domestication status	Row type	Growth habit	Origin
Morex	Cultivar	6-rowed	Spring	USA
Barke	Cultivar	2-rowed	Spring	Germany
Igri	Cultivar	2-rowed	Winter	Germany
BCC131	Landrace	6-rowed	Spring	Morocco
HOR1969	Landrace	Intermedium		Tibet
FT11	Wild	2-rowed	Facultative	Israel (desert)
FT67	Wild	2-rowed	Facultative	Israel (coast)
FT279	Wild	2-rowed	Facultative	Afghanistan
FT581	Wild	2-rowed	Facultative	Turkey

The passport data are according to Russell *et al.* (2016).

### Data quality

Most samples mapped to the barley reference sequence at a high rate (≥80%), but eight samples (all from genotype BCC131) had a mapping rate of <80% (Supplementary Figs S3, S4). Six of these had a mapping rate between 50% and 79%, and one sample had a mapping rate of 30%. To determine the cause of the low mapping rate of the eight samples, a Basic Local Alignment Search Tool (BLAST) run was conducted. For those samples with a mapping rate between 50% and 79%, the source of contamination is the *Barley stripe mosaic virus* (BSMV; Supplementary Fig. S4), while the sample with the lowest mapping rate (30%) is contaminated with human DNA (Fig. S5). BCC131 samples were included in our analyses anyway because the effect of sequence contamination, reduced coverage, merely reduces statistical power for variant calling. While this reduction decreases power for ASE and analysis of total transcript abundance, the data for genes that remain informative are still useful.

### Principal component analysis

**Fig. 2.**
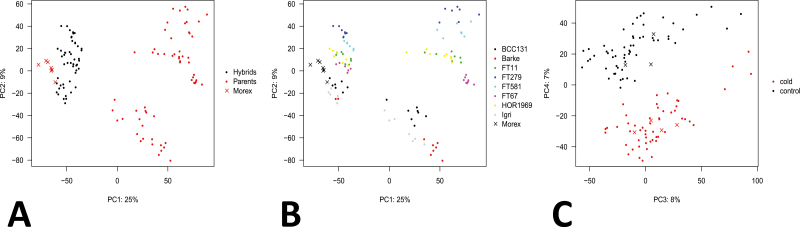
Principal component analysis. (A) and (B) are the same figure but colored differently in order to highlight different aspects of the plot. (A) Principal component 1 (PC1) separates samples based on generation. Parental samples cluster on the right, while hybrids cluster on the left, closer to the common reference parent, Morex. (B) PC2 separates samples based on accession. The hybrids highlighted in (A) are colored in (B) to show which paternal parent they correspond to since Morex is the common maternal parent for all hybrids. (C) PC4 separates samples according to treatment. The proportions of variance explained by each PC are indicated on the axis labels.

After checking our gene expression data quality, we examined the data to see if they match our expectations to ensure that they are reliable. PCA was conducted using kallisto-derived expression data. Kallisto is capable of outputting both normalized reads (transcripts per million; TPM) and count data. Our analysis was conducted using count data. The first principal component explains 25% of the variance and separates the parental genotypes from Morex, the common maternal parent for all hybrids. The hybrids cluster between Morex and the parents, as expected for hybrids (Fig. 2A). The second principal component explains 9% of the variance. Three parental samples (BCC131, Barke, and Igri) form a cluster separate from the other accessions. The cultivars Barke and Igri are from Germany and BCC131 is a Moroccan landrace, while wild barleys FT11 and FT67 originate from different environments in Israel, FT279 is from Afghanistan, FT581 is from Turkey, and the landrace HOR1969 is from Tibet (Fig. 2B). The third principal component explains 8% of the variance. Samples along this component cluster by accession; however, only HOR1969 loosely clusters separately from the others (Supplementary Fig. S6). The fourth principal component explains 7% of the variance and separates samples according to treatment (Fig. 2C). The PCA results show that samples cluster according to the principal factors in our experiment (i.e. generation, genotype, and treatment). Therefore, the data may be used to confidently determine ASE.

### Exome capture and SNP calling

The PCA described above was conducted using overall transcript abundance as estimated with kallisto, but these results are for overall expression and are not allele specific. HISAT2 was used for variant-aware alignment of reads in order to determine from which allele a transcript originated. For high-confidence SNPs, ascertained for each cross in exome capture data for both inbred parents and one hybrid individual, we collected exome capture data of the hybrids and used previously published exome capture of the parents (Russell *et al.*, 2016). Exome capture mapping statistics are presented in Supplementary Fig. S7. By comparing SNPs between parental accessions and confirming these SNPs in exome capture from hybrids between these accessions and transcript (RNA) data, we were able to determine allele-specific transcript abundance in the hybrids for those genes that have sequence differences between the parents. The numbers of informative SNPs and genes are presented in Table 2. SNPs are informative if they reside in genic regions since SNPs are only useful for ASE when they are transcribed. SNPs in regulatory regions are important for ASE, but they cannot be detected from RNA-seq data. The number of informative genes for BCC131 (2589) is lower than expected based on the other landrace, HOR1969 (6850 genes), as a result of lower coverage due to contamination as discussed above (Supplementary Figs S3–S5). Otherwise, the general pattern of wild accessions being more diverged from Morex (8282–9318 informative genes) compared with cultivars (4296 and 4634 genes for Barke and Igri, respectively) is, unsurprisingly, observed.

**Table 2.** The number of informative SNPs, the number of informative genes, and the percentage of total high-confidence genes in the barley genome between each accession relative to the cultivar Morex

	Barke	Igri	BCC131	HOR1969	FT11	FT67	FT279	FT581
No. of informative SNPs	16 716	14 905	7874	21 593	27 436	24 854	24 418	26 650
No. of informative genes	4926	4634	2589	6850	9318	8590	8282	8940
% Total genes	12.40	11.66	6.52	17.24	23.45	21.62	20.84	22.50

### Assignment of genes to regulatory categories

**Fig. 3.**
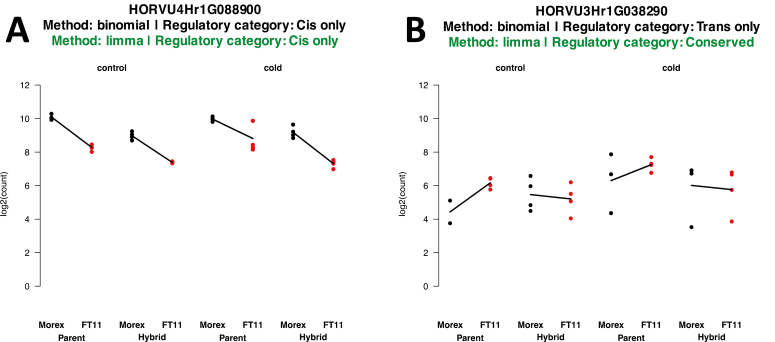
Example profiles for two genes illustrate the effect of the statistical differences between the binomial testing and linear model methods. Both methods agree in (A) because of the similar expression values between replicates; however, in (B), the large differences in expression between replicates mean that confidence in the true expression value is low.

**Fig. 4.**
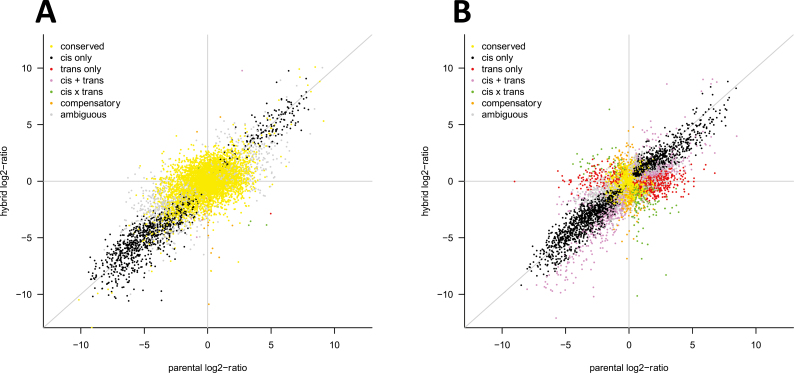
A global view of regulatory category assignment for one hybrid and its parents, in this case Morex and FT11. The *x*-axis shows the log_2_ ratio of expression difference between the parents, while the *y*-axis shows the log_2_ ratio of expression difference between the parental alleles in the hybrid. (A) The global view using the linear model method; (B) the method used by McManus *et al.* (2010).

For each of the informative genes, we mapped transcripts to determine whether or not there was ASE. Initially, we followed the methods used by McManus *et al.* (2010); however, as we inspected expression plots further, we realized that genes assigned to the *trans* only category differed greatly in their expression levels between replicates (Fig. 3B). Use of the linear model resulted in a drastic reduction in the number of genes with *trans* effects including *trans* only, *cis*+*trans*, and *cis*×*trans* (Fig. 4A; Table 3) compared with the binomial method used by McManus *et al.* (2010) (Fig. 4B; Table 4). This is in line with what other authors have found in other organisms (Goncalves *et al.*, 2012; Osada *et al.*, 2017). Another notable trend is that the number of genes assigned to the conserved class of regulatory variation is higher when using a linear model. Approximately 80% of the total number of genes were assigned to this class using a linear model versus ~20% using the binomial/Fisher’s exact test (Tables 3, 4). Our results might be an underestimation of the amount of *cis* regulation in barley, but this is likely to be a consequence of the stricter threshold we used to declare allelic imbalance and hence a lower number of genes showing allelic differences in expression. In addition, regulation of gene expression appears to be stable in response to environmental stress, consistent with the findings of Cubillos *et al.* (2014). Regulatory category plots for all crosses are given in Supplementary Fig. S8.

**Table 3.** Regulatory category assignments of genes using the linear model (limma) method

Category	Treatment	Barke	Igri	BCC131	HOR1969	FT11	FT67	FT279	FT581
*Cis* only	Control	283	340	368	811	1065	962	894	953
	Cold	8	230	352	782	748	904	967	1033
	Intersection	8	172	289	617	641	754	749	789
*Trans* only	Control	0	0	3	3	1	3	2	4
	Cold	0	0	1	1	3	0	1	1
	Intersection	0	0	0	1	0	0	0	0
*Cis*+*trans*	Control	0	0	3	15	7	14	15	12
	Cold	0	0	1	9	7	14	18	18
	Intersection	0	0	1	7	3	9	12	6
*Cis*×*trans*	Control	0	1	3	1	5	0	3	9
	Cold	0	1	2	0	1	0	3	4
	Intersection	0	1	1	0	1	0	1	2
Compensatory	Control	0	3	35	22	29	17	28	29
	Cold	0	1	39	25	29	16	29	30
	Intersection	0	1	28	17	20	12	19	20
Conserved	Control	3969	3924	1906	5428	7278	6704	6610	7024
	Cold	3960	3916	1938	5514	7871	6945	6654	7093
	Intersection	3738	3713	1771	5165	7047	6408	6241	6582
Ambiguous	Control	674	366	271	570	933	890	730	909
	Cold	958	486	356	519	659	711	610	761
	Intersection	454	159	87	179	229	300	222	283
Total	Control	4926	4634	2589	6850	9318	8590	8282	8940
	Cold	4926	4634	2589	6850	9318	8590	8282	8940

**Table 4.** Regulatory category assignments of genes using the binomial and Fisher’s exact test method of McManus *et al.* (2010).

Category	Treatment	Barke	Igri	BCC131	HOR1969	FT11	FT67	FT279	FT581
*Cis* only	Control	1459	1118	759	1738	2704	2546	2338	2303
	Cold	1084	1294	749	1924	2073	2458	2239	3070
	Intersection	744	502	413	983	1041	1457	1134	1565
*Trans* only	Control	287	448	139	1174	1120	1116	1075	731
	Cold	274	462	241	851	863	821	1164	692
	Intersection	41	84	26	319	249	265	283	142
*Cis*+*trans*	Control	241	806	248	1012	1110	969	1082	580
	Cold	158	472	228	763	1262	586	1430	741
	Intersection	70	292	101	416	459	308	618	251
*Cis*×*trans*	Control	77	173	107	296	285	246	298	235
	Cold	48	143	117	241	311	176	344	189
	Intersection	14	29	39	94	100	69	100	50
Compensatory	Control	89	291	200	212	272	193	247	313
	Cold	40	168	90	208	467	154	219	191
	Intersection	5	24	12	34	44	23	25	22
Conserved	Control	1173	909	653	1170	1995	1578	1617	2620
	Cold	968	925	623	1417	2458	2127	1280	1932
	Intersection	469	354	319	526	1068	889	556	1058
Ambiguous	Control	1600	889	483	1248	1832	1942	1625	2158
	Cold	2354	1170	541	1446	1884	2268	1606	2125
	Intersection	992	330	144	408	541	725	506	670
Total	Control	4926	4634	2589	6850	9318	8590	8282	8940
	Cold	4926	4634	2589	6850	9318	8590	8282	8940

The numbers of genes in each regulatory category are roughly similar for control samples and those in response to environmental stress, but, since these tests were conducted independently, we wanted to know how similar these lists are. To answer this question, we found the intersection of gene lists for each comparison (Table 3, 4). The results show that regulatory category assignments are robust to environmental stress, especially for genes with conserved regulation. On average, 94% (~90–96%) of genes in this category are present in both treatments for all crosses. Since it appears that results for category assignments are similar between treatments, we wanted to know if we could detect more *trans* effects by considering control and cold treatments together to obtainn additional replicates, in order to gain statistical power within the linear model. The results (Table 5; Supplementary Fig. S8) are similar to when each treatment was analyzed separately (Table 3). A moderate increase in the number of *trans*, *cis*+*trans*, and *cis*×*trans* effects was observed, but not to the same extent as found by McManus *et al.* (2010).

**Table 5.** Regulatory category assignments for each cross when treatments were not considered separately and instead grouped as additional replicates

	Barke	Igri	BCC131	HOR1969	FT11	FT67	FT279	FT581
*Cis* only	178	584	466	1130	1249	1181	1282	1291
*Trans* only	2	0	0	1	7	0	3	0
*Cis*+*trans*	1	1	5	18	20	15	36	30
*Cis*×*trans*	0	4	2	3	4	0	3	4
Conserved	3938	3641	1819	5081	7145	6621	6293	6770
Compensatory	0	13	34	28	44	19	30	41
Ambiguous	896	391	263	589	849	826	635	804
Total	5015	4634	2589	6850	9318	8662	8282	8940

The linear model was used to generate these results.

### Expression of known cold-responsive genes

**Fig. 5.**
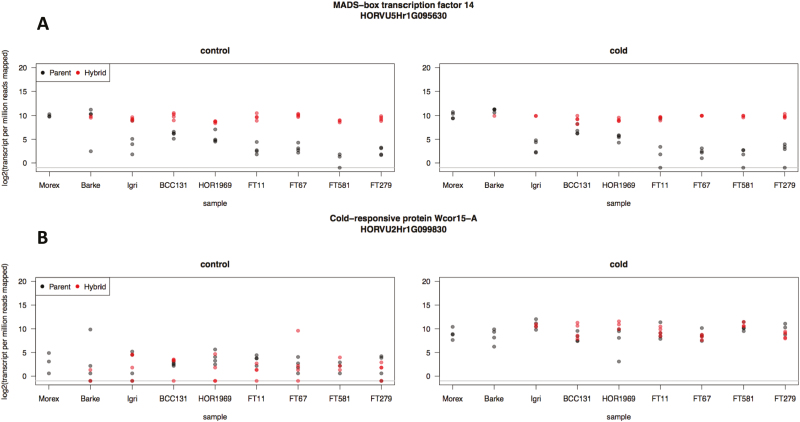
Expression (log_2_-transformed transcripts per million reads mapped) values for parents (black) and hybrids (red) from each sample: (A) *VRN1* (HORVU5H1rG095630) is expressed at higher levels in spring types (which do not require vernalization) than the winter type, landraces, and wild barleys. The hybrids show this same higher level of *VRN1* expression, indicating that the Morex allele is dominant. (B) *COR14B* (HORVU2Hr1G099830) shows a response to chilling in the cold-treated samples, also as expected.

In addition to looking at general expression patterns, we are also interested in the expression of known cold-responsive genes. Therefore, we looked into the expression patterns of these genes including *Vernalization1* (*VRN1*) and *Cold-Regulated 14B* (*COR14B*). The expression of both *VRN1* (HORVU5Hr1G095630) and *COR14B* (HORVU2Hr1G099830) matched our expectations. Morex and Barke (spring types) have higher expression levels of *VRN1* than Igri (a winter type), both landraces, as well as all wild accessions (Fig. 5A). Expression of *VRN1* is maintained at low levels in wild and winter barleys until it has endured a prolonged period of cold exposure, or vernalization. This vernalization requirement is evolutionarily advantageous because flowering will only occur when prevailing environmental conditions are favorable. For comparison, we also show the expression pattern of a developmental gene (*Ppd-H1*; involved in the photoperiod response) in Supplementary Fig. S9.

Hybrids have *VRN1* expression levels that match those of the spring types, demonstrating that the loss of a vernalization requirement is dominant. The expression profiles are similar for both control and cold treatments, which is also expected since *VRN1* expression only increases after several weeks of exposure to cold temperatures and our samples were only exposed to cold for 3 h. The duration of exposure to cold was chosen because the chilling response to cold occurs rapidly in barley (Cattivelli and Bartels, 1989) and we were interested in the initial rapid response to stimulus (cold) rather than the long-term adaptation to cold temperatures/low temperature tolerance as Cubillos *et al.* (2014) did with adaptation to mild drought stress. The expression of *VRN1* in the hybrid confirms that the hybrid shows the correct inheritance patterns. The cold-responsive gene *COR14B*, however, shows a clear increase in response to cold treatment (Fig. 5B).

### Dominant versus additive inheritance

In addition to regulatory categories discussed earlier, we are also interested in examining the mode of inheritance of genes in our data set. Many genes exhibit Mendelian inheritance (dominance versus recessive). However, many other genes exhibit quantitative or additive inheritance. Still other inheritance modes (heterosis) also exist. Heterosis, also known as hybrid vigor, occurs when expression of a gene is outside the range of the parental values (i.e. overdominance). We were interested in exploring the distribution of these inheritance modes in our data. The summary of the modes of inheritance is reported in Table 6 for control samples and in Table 7 for cold samples. Numbers are unavailable for Morex×Barke under the cold treatment because of a lack of replicates for cold Barke hybrid samples. Relatively few genes show heterotic effects (overdominance) under both control and cold conditions. For most crosses, these categories represent <1% of differentially expressed genes. Under no circumstance did heterosis affect >2% of differentially expressed genes. Approximately one-third of all differentially expressed genes have additive effects under both conditions. (25.5–37.8% control and 30.3–38.0% cold). Genes showing dominance together represent about another third of differentially expressed genes. In nearly every cross, more Morex alleles are dominantly expressed than the paternal allele. This could be an effect of Morex being the maternal allele, but it could also reflect a tendency of domesticated alleles to be more highly expressed than wild alleles. Of course, Morex is also the reference genome, so it is likely that there is some bias towards the reference allele. The one case where the paternal allele has more dominantly expressed alleles than Morex involved Igri, a winter cultivar, under control conditions. Otherwise, the trend seems to be that the numbers of dominant genes are more equally distributed between the two parents for cultivars (Barke and Igri) and landraces (BCC131 and HOR1969) than for the wild accessions (FT11, FT67, FT279, and FT581). Another quarter to one-third (27.4–36.2% control and 24.5–34.3% cold) of all differentially expressed genes were placed into the ambiguous category, and a handful of others did not fit into any of the other categories. It is difficult to speculate which category these genes truly belong to. We might assume that they would fall into one of the main three categories (additive, Morex dominant, or paternal allele dominant) in a proportional manner, but we cannot state this with certainty.

**Table 6.** Mode of inheritance assignment counts for each cross under control conditions

Category	Barke	Igri	BCC131	HOR1969	FT11	FT67	FT279	FT581
Additive	1722 (25.5%)	1960 (34.0%)	2542 (37.8%)	2143 (30.8%)	2849 (35.2%)	2473 (31.1%)	2837 (37.2%)	2949 (35.4%)
Morex dominant	1648 (24.4%)	811 (14.1%)	890 (13.2%)	1446 (20.8%)	1406 (17.3%)	1516 (19.1%)	1425 (18.7%)	1826 (21.9%)
Paternal allele dominant	756 (11.2%)	1115 (19.3%)	832 (12.4%)	976 (14.0%)	1046 (12.9%)	954 (12.0%)	1219 (16.0%)	1132 (13.6%)
Morex over dominant	125 (1.8%)	16 (0.3%)	11 (0.2%)	12 (0.2%)	11 (0.1%)	28 (0.4%)	12 (0.2%)	74 (0.9%)
Paternal allele over dominant	7 (0.10%)	5 (0.09%)	3 (0.04%)	7 (0.1%)	3 (0.04%)	4 (0.05%)	5 (0.07%)	3 (0.04%)
Ambiguous	2327 (34.4%)	1828 (31.7%)	2441 (36.2%)	2336 (33.6%)	2759 (34.0%)	2877 (36.2%)	2095 (27.5%)	2278 (27.4%)
Not assigned	174 (2.6%)	30 (0.5%)	15 (0.2%)	31 (0.4%)	30 (0.4%)	101 (1.3%)	32 (0.4%)	59 (0.7%)
Total	6759	5765	6734	6951	8104	7953	7625	8321

Percentage values may not add up to exactly 100% due to rounding.

**Table 7.** Mode of inheritance assignment counts for each cross under cold (4 °C) conditions

Category	Barke	Igri	BCC131	HOR1969	FT11	FT67	FT279	FT581
Additive	NA	1745 (30.3%)	2557 (38.0%)	2257 (32.5%)	2984 (36.8%)	2691 (33.8%)	2591 (34.0%)	2935 (35.3%)
Morex dominant	NA	1239 (21.5%)	962 (14.3%)	1462 (21.0%)	1514 (18.7%)	1596 (20.1%)	1836 (24.1%)	2030 (24.4%)
Paternal allele dominant	NA	1057 (18.3%)	867 (12.9%)	997 (14.3%)	992 (12.2%)	1167 (14.7%)	1205 (15.8%)	1097 (13.2%)
Morex over dominant	NA	62 (1.1%)	32 (0.5%)	32 (0.5%)	32 (0.4%)	55 (0.7%)	106 (1.4%)	111 (1.3%)
Paternal allele over dominant	NA	5 (0.09%)	3 (0.04%)	7 (0.1%)	3 (0.04%)	38 (0.5%)	5 (0.07%)	3 (0.04%)
Ambiguous	NA	1646 (28.6%)	2307 (34.3%)	2188 (31.5%)	2575 (31.8%)	2401 (30.2%)	1871 (24.5%)	2136 (25.7%)
Not assigned	NA	11 (0.2%)	6 (0.09%)	8 (0.1%)	4 (0.05%)	5 (0.06%)	11 (0.1%)	9 (0.1%)
Total	NA	5765	6734	6951	8104	7953	7625	8321

Values for Barke are unavailable because of a lack of replicates from hybrid in the cold treatment. Percentage values may not add up to exactly 100% due to rounding.

## Discussion

We are interested in understanding the effect of domestication on patterns of gene expression and regulatory variation in barley. To accomplish this, we combined the use of ASE on a small panel of wild and domesticated barleys and their F_1_ hybrids with a cold stress treatment according to established methods (Cowles *et al.*, 2002; Cubillos *et al.*, 2014; Lemmon *et al.*, 2014). Several lines of evidence indicate that the approach worked and our results are reliable. First, samples cluster according to generation (Fig. 2A), accession (Fig. 2B), and treatment (Fig. 2C). The expression profiles of cold-responsive genes such as *VRN1* and *COR14B* also behave as expected (Fig. 5A, B). Of 39 734 high-confidence genes in the barley genome, we were able to quantify ASE for between 2589 (BCC131) and 8940 (FT581) genes (Table 2). We cannot measure ASE for genes that lack SNPs because it is impossible to unambiguously assign such transcripts to a parental allele without at least one SNP to verify the allele of origin. Other genes may not be expressed at sufficient levels to have statistical power for ASE. Based on previous studies (McManus *et al.*, 2010; Lemmon *et al.*, 2014), we expected to find a similar number of genes regulated in *cis* and *trans*; however, we found almost a complete absence of genes regulated in *trans*. The increased expression of cold-responsive genes (*COR14B*, Fig. 5B) after cold treatment suggests that the cold treatment induced TFs to elicit a response to cold. Since TFs act in *trans*, some *trans* effects are expected; however, a small number of TFs may be more plausible than hundreds or thousands of *trans*-acting genes observed in earlier studies, to minimize pleiotropic effects (West *et al.*, 2007). In general, genes with *trans* effects may not cause pleiotropic effects if they do not disrupt highly connected nodes in a network (Jeong *et al.*, 2001; Fraser *et al.*, 2002). Further, TFs do not necessarily cause large pleiotropic effects. Work in *Caenorhabditis elegans* shows that mutations in the Ras signaling pathway that activate multiple TFs are more deleterious than mutations affecting only TFs (Kayne and Sternberg, 1995). In our present study, the genes regulated in *trans* according to the linear model do not appear to have any great significance. The expression levels of these genes are low and are plagued with missing data (e.g. some of the genes are expressed in one genotype, but not another) and annotations are ambiguous. It is also possible that the parameters of our analysis are too strict, resulting in false negatives; however, other studies have probably suffered from false positives. Clearly, a method is needed that rejects *trans* effects that are truly absent, but accepts real *trans* effects.

Evidence for regulatory changes in response to environmental stress is absent from our data, in agreement with Cubillos *et al.* (2014). However, we cannot rule out that the use of a different environmental stress (high temperature, drought, or salinity) could induce a more variable response. Cubillos *et al.* (2014) also found that roughly half of the genes in their samples had compensatory effects, meaning that *cis* and *trans* effects are opposite. In contrast, we found that half of our genes had conserved effects. In addition, Cubillos *et al.* (2014) observed an increase in the number of genes with *trans* effects that resulted in a change in direction in response to the environment, rather than a change in magnitude, compared with genes with *cis* effects. We were not able to make such a comparison, since genes with *trans* effects are virtually absent in our data set.

Wittkopp *et al.* (2008) found a greater amount of *cis*-regulatory expression differences between species rather than within species, which could also explain why *trans* effects were more pronounced in studies that examined expression differences between *Drosophila* species (McManus *et al.*, 2010). However, Osada *et al.* (2017) also noted large variances in their samples; therefore, our hypothesis that differences observed for *trans* regulation are likely to be false positives as a result of statistical artifacts seems to be plausible.

The observation of a greater number of *cis*- compared with *trans*-acting factors has important implications for the use of crop wild relatives in plant breeding. Insights into gene regulation in barley such as this will help to exploit wild genetic resources in elite germplasm (Schmalenbach *et al.*, 2009). In nature, it appears that *cis* effects preferentially accumulate, probably due to fewer pleiotropic effects compared with *trans* effects (Prud’homme *et al.*, 2007). Similarly, in plant breeding, genetic background is known to influence the expression of genes due to epistatic interactions (Kroymann and Mitchell-Olds, 2005; Blanc *et al.*, 2006). For novel quantitative trait loci (QTLs) introgressed into elite germplasm to be useful, the beneficial trait must be expressed in the elite background. Genes regulated in *cis* will be more likely to be expressed at the same level in a novel background as in their native background when their regulatory sequence is co-inherited due to linkage, whereas co-inheritance of *trans* regulators will occur less frequently due to independent segregation. Introgression of a gene as well as its *trans* regulator would be complicated enough, but could also have deleterious effects in the new genetic background if the *trans* regulator epistatically affects the expression of off-target genes. The recipient background may also regulate the introgression through *trans* regulators. One way to study this experimentally is to use near-isogenic lines (NILs) that contain as many of the total possible genes in small introgressions throughout the genome. Guerrero *et al.* (2016) conducted such an experiment in tomato. They showed that introgressed genes tend to be down-regulated while native (non-introgressed) regions tend to be up-regulated. The authors concluded that c*is* and *trans* regulation have roughly equal contributions to expression divergence.

The *cis*-regulatory regions of genes can be large, extending for thousands of kilobases such as the case with *Teosinte Branched 1* (*tb1*) in maize, which has at least one regulator from 58 kb to 69 kb upstream from the 5' start site (Clark *et al.*, 2006). Therefore, it is possible that recombination may occur between a *cis*-regulatory sequence and the gene it controls. However, *cis*-regulatory regions are not well defined. This possibility highlights one limitation of the applications of our study. Due to our experimental design, we can only infer the presence and relative contribution of *cis*- or *trans*-acting regulation, but we cannot map these regulators; therefore, we do not know the genomic position of these regulators. An experimental approach known as expression quantitative trait locus (eQTL) mapping allows gene expression to be mapped as quantitative traits in experimental populations or by association genetics. These studies allow for mapping of regulatory elements; however, it is still not always clear at what distance threshold an eQTL would be acting in *cis* or in *trans*, since these distance thresholds are often arbitrary (Lagarrigue *et al.*, 2013). In addition, eQTLs are more properly referred to as local or distant, rather than *cis* or *trans* (Lagarrigue *et al.*, 2013). These studies are also more difficult and expensive because they require a large mapping population to be both genotyped and assayed for genome-wide expression values.

Alignment bias due to polymorphism or structural variants is a well-documented problem with ASE studies (Degner *et al.*, 2009; Stevenson *et al.*, 2013) and our data set is no exception due to the use of a single genotype (Morex) as a reference. We see bias towards the Morex allele in each of our crosses. In Supplementarry Fig. S10, we show two example plots. In part due to these limitations, a single reference genotype is no longer considered to be sufficient to capture the full diversity present in a given species. The concept of the pan-genome posits that any species has a set of genes present in all accessions (the core genome), genes that are present in some, but not all accessions (the dispensable genome), and lineage-specific genes that are only present in a single accession. In this context, additional reference genomes are needed. Other barley genotypes, such as Barke and FT11, are not available at present. There is a barley pan-genomic project underway, at which point these genotypes and others will be available (Monat *et al.*, 2019). For now, it is necessary to interpret our results with caution. When the genomes of these other accessions do become available, it will become possible to re-analyze these data to measure the impact of the reference bias. One possible re-analysis method has already been conducted in *Drosophila melanogaster*. Graze *et al.* (2012) and Fear *et al.* (2016) used multiple *D. melanogaster* assemblies to build a combined reference to use for ASE analysis. The result is a near-complete elimination of mapping bias. Once additional barley reference assemblies are available, we can perform the same analysis.

The availability of additional reference genomes will also allow for re-analysis of these data with a Bayesian approach that allows for direct comparison of environmental effects (León-Novelo *et al.*, 2018). Additional reference genomes are necessary because the method incorporates the number of RNA reads which align equally well to both parental genomes.

The results of this experiment may be useful in advancing the understanding of heterosis in barley. Autogamous crops such as wheat and barley have yet to realize the types of gains that have been achieved in allogamous crops such as maize. These gains may be attributed to hybrid vigor, also known as heterosis. Most work done to date in wheat (Zhao *et al.*, 2015; Jiang *et al.*,2017) and barley (Mühleisen *et al.*, 2013; Phillipp *et al.*, 2016) has been quantitative in nature. Our work contributes knowledge about the molecular basis of hybrid vigor that can complement the quantitative work.

## Supplementary data

Supplementary data are available at *JXB* online.

**Table S1.** Accession numbers for individual accessions deposited in the European Nucleotide Archive (ENA).

**Table S2.** Gene category assignment for Barke×Morex.

**Table S3.** Gene category assignment for Igri×Morex.

**Table S4.** Gene category assignment for BCC131×Morex.

**Table S5.** Gene category assignment for HOR1969×Morex.

**Table S6.** Gene category assignment for FT11×Morex.

**Table S7.** Gene category assignment for FT67×Morex.

**Table S8.** Gene category assignment for FT279×Morex.

**Table S9.** Gene category assignment for FT581×Morex.

**Fig. S1.** Expected relative expression levels for *cis* only effects, *trans* only effects, *cis+trans*, and *cis×trans*. 

**Fig. S2.** Geographical distribution of wild barleys used in this study (except FT279 from Afghanistan, which is not in the frame); and Principal component analysis based on exome capture data from Russell *et al.* (2016) that was the basis of selection of parents for use in this study.

**Fig. S3.** HISAT mapping rate.

**Fig. S4.** *Barley stripe mosaic virus* (BSMV) kallisto versus HISAT mapping rate.

**Fig. S5.** Basic Local Alignment Search Tool (BLAST) results for the forward read of Sample_B_088 (Cold_BCC131_H3).

**Fig. S6.** PCA plot of PC3 and 4. 

**Fig. S7.** Exome capture mapping statistics for the eight hybrids used in this study.

**Fig. S8.** Log_2_ ratio plots of parents (*x*-axis) versus parental alleles in the hybrid (*y*-axis) for all crosses when treatments were not considered separately and instead grouped as additional replicates.

**Fig. S9.** Expression (log_2_-transformed transcripts per million reads mapped) values for parentsand hybrids from each sample for *Ppd-H1* (HORVU2Hr1G013400). 

**Fig. S10.** Distribution of log_2_ fold change values for two crosses, Morex×Igri and Morex×FT11.

eraa036_suppl_Supplementary_Figures_S1-S8

eraa036_suppl_Supplementary_Tables_S1-S9
