# Supplementary material for: The contribution of cis- and trans-acting variants to gene regulation in wild and domesticated barley under cold stress and control conditions
Source: J Exp Bot. 2020 Jan 28;71(9):2573–84. doi: 10.1093/jxb/eraa036 (PMC7210754; doi:10.1093/jxb/eraa036)
Supplement: eraa036_suppl_Supplementary_Figures_S1-S8 [file eraa036_suppl_supplementary_figures_s1-s8.pdf]

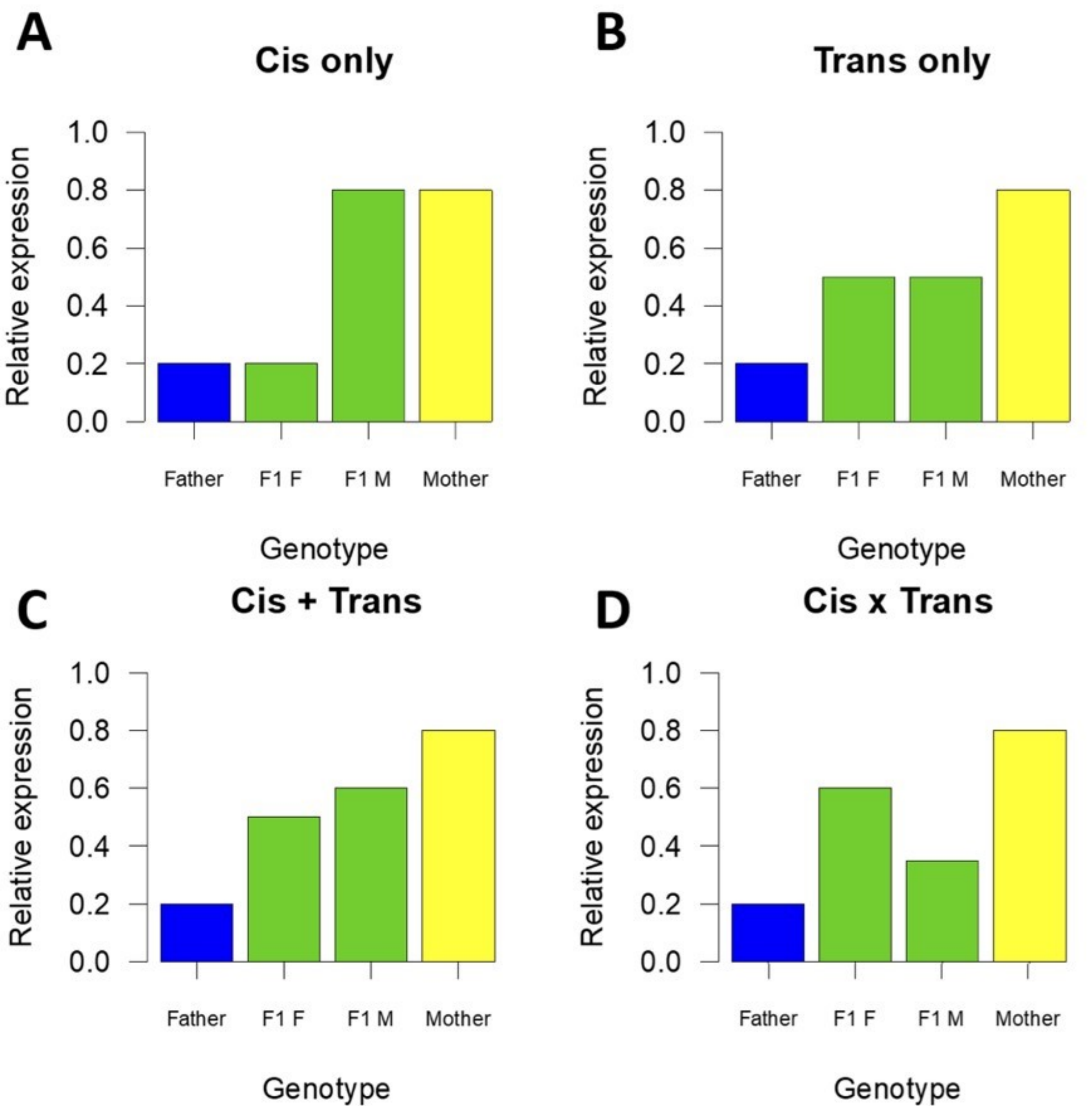

Figure S1. Expected relative expression levels for A) *Cis* only effects, B) *Trans* only effects, C) *Cis + Trans* and D) *Cis × Trans*. Bar plots for the father (blue) and mother (yellow) are the result of the combined effects of both alleles in the respective accession. Two green bars (middle) each represent a single allele in the hybrid individual. F1 F is the hybrid allele derived from the father while F1 M is the hybrid allele derived from the mother.

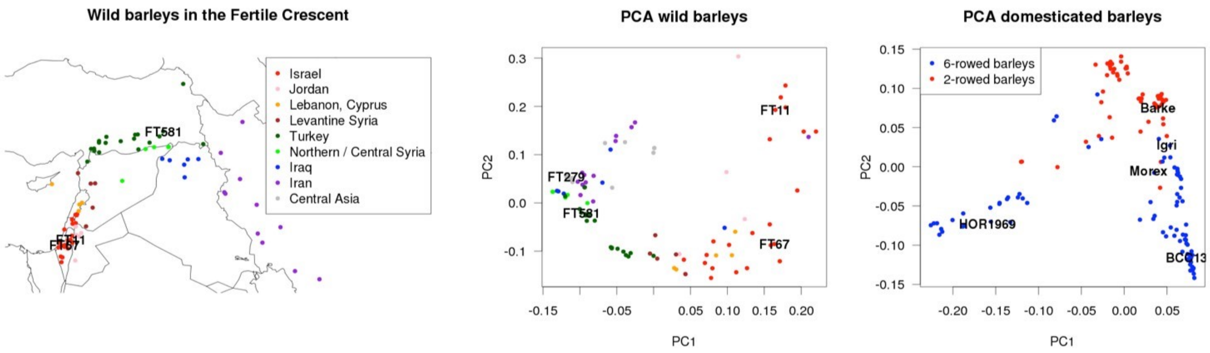

Figure S2. Geographical distribution of wild barleys used in this study (except FT279 from Afghanistan, which is not in the frame); and Principal component analysis based on exome capture data from (Russell et al. 2016) that was the basis of selection of parents for use in this study.

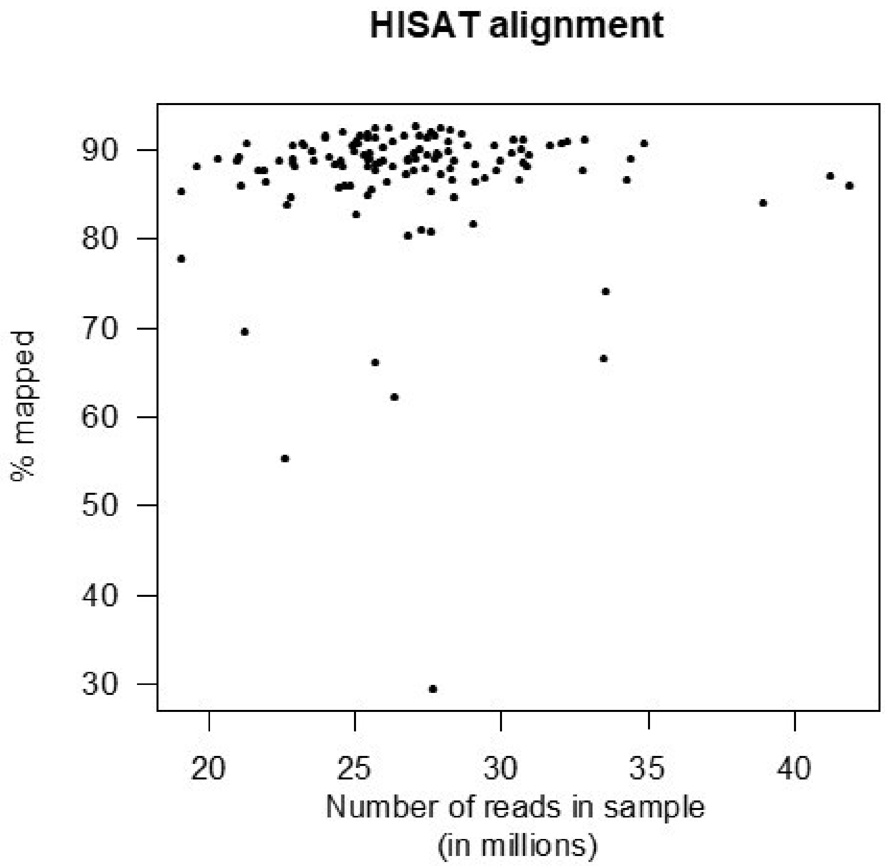

Figure S3. HISAT mapping rate.

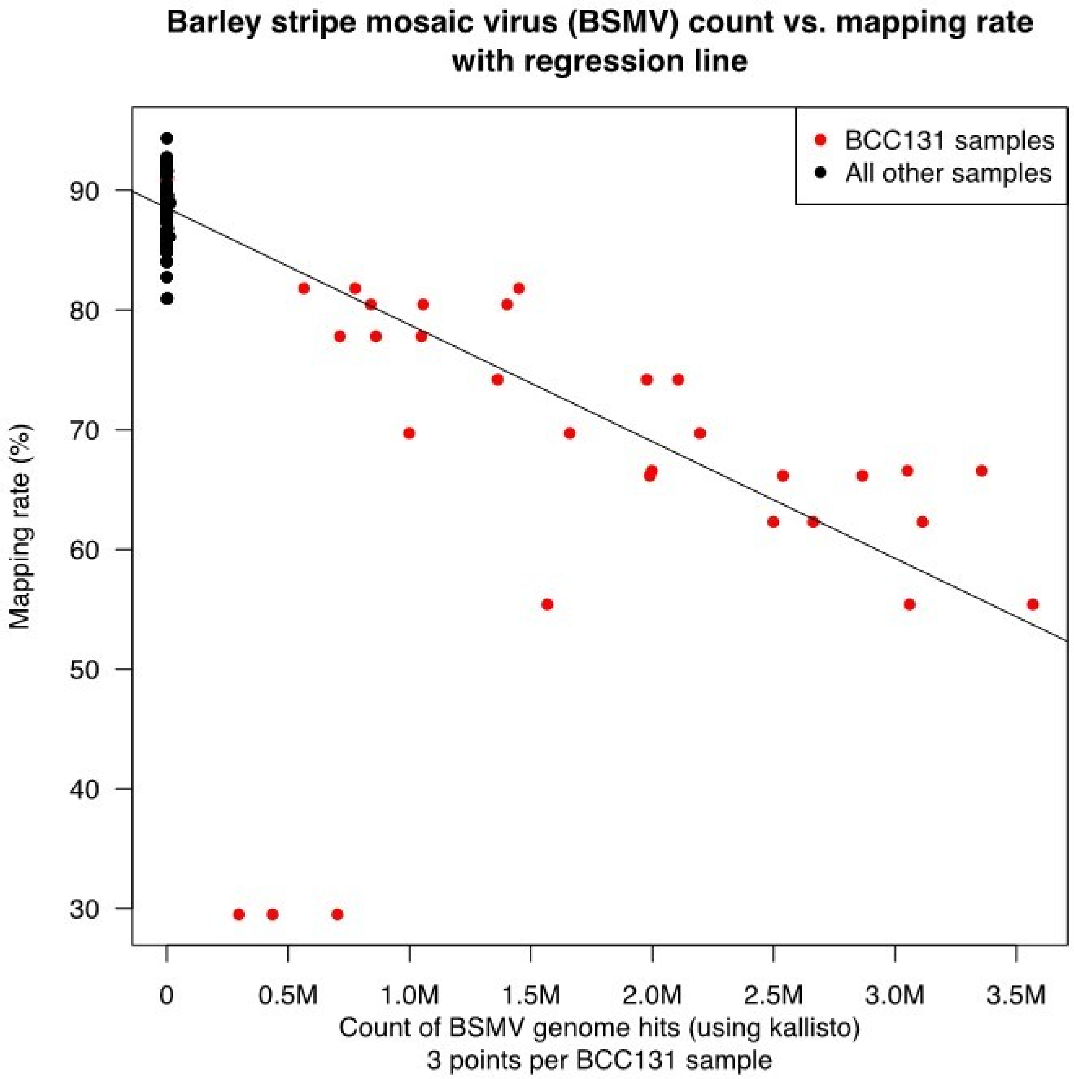

Figure S4. Barley stripe mosaic virus (BSMV) kallisto vs. HISAT mapping rate.

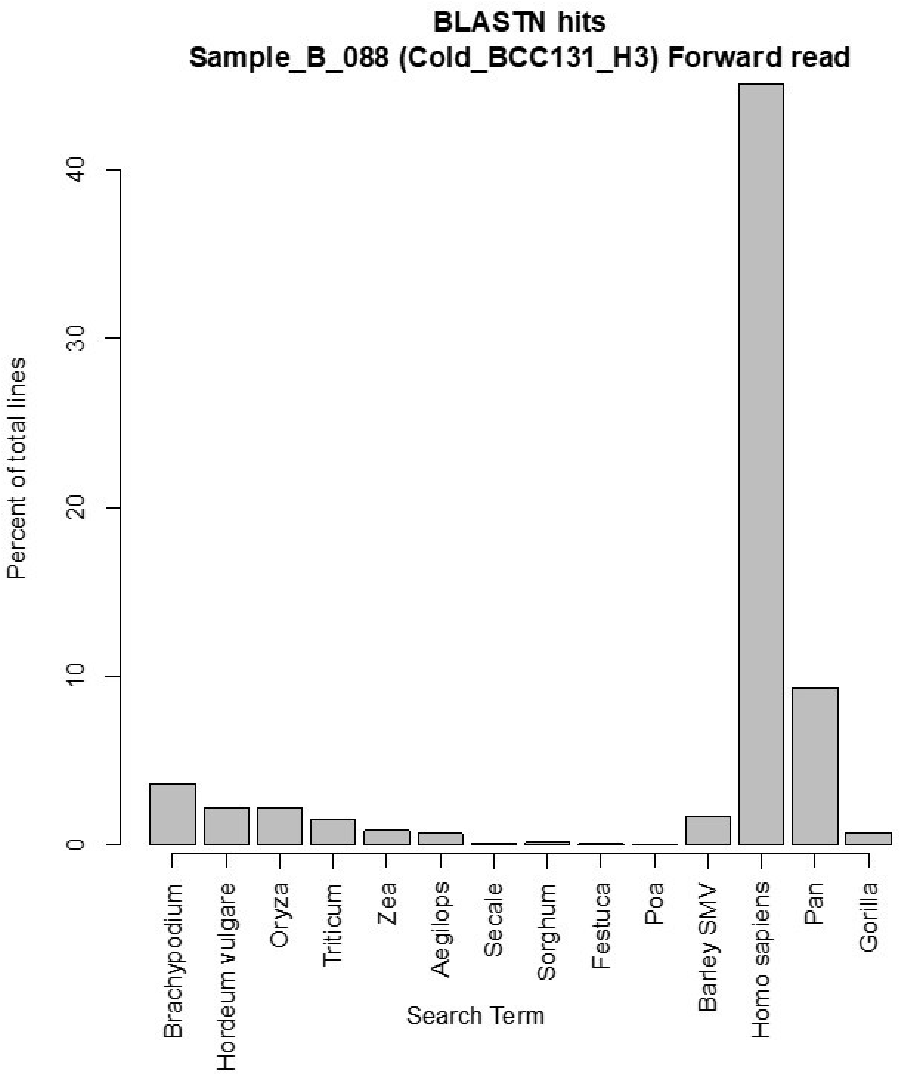

Figure S5. Basic Local Alignment Search Tool (BLAST) results for the forward read of Sample_B_088 (Cold_BCC131_H3).

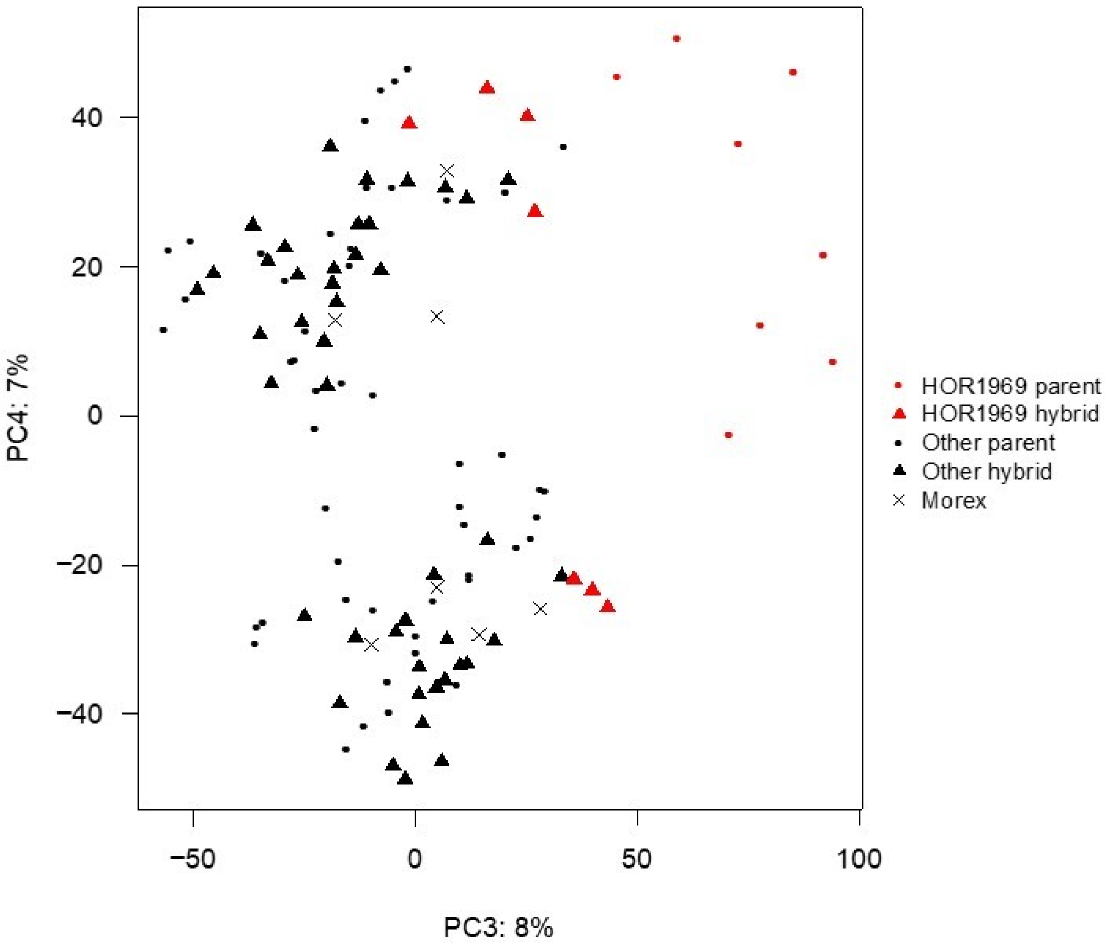

Figure S6. PCA plot of PC3-4. The plot is identical to the one presented in Figure 2C except that HOR1969 samples are colored in red and all other samples are colored in black. Sample shapes designate generation. Circles are parental samples while hybrids are triangles. Morex is indicated with a "×" symbol.

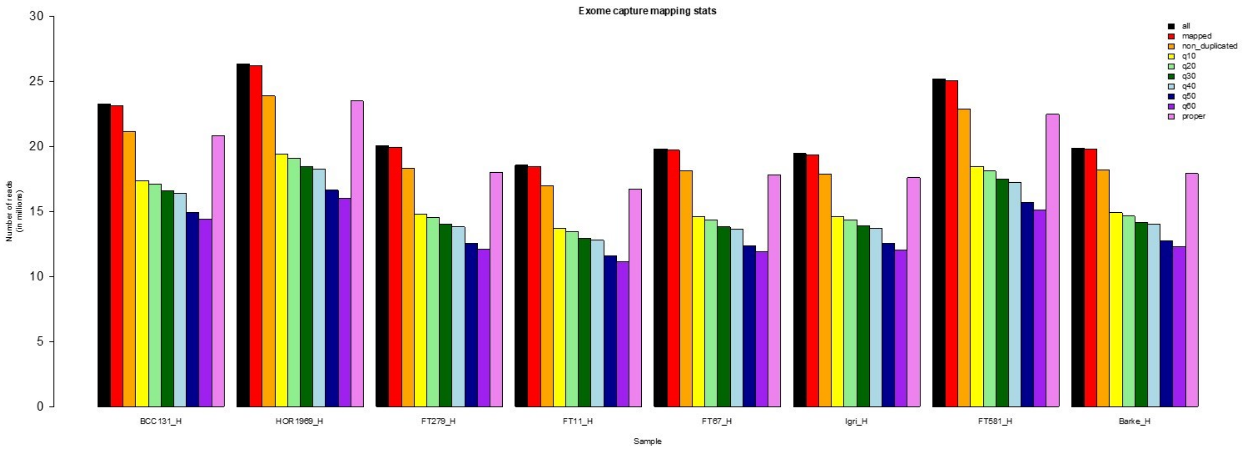

Figure S7. Exome capture mapping statistics for the eight hybrids used in this study.

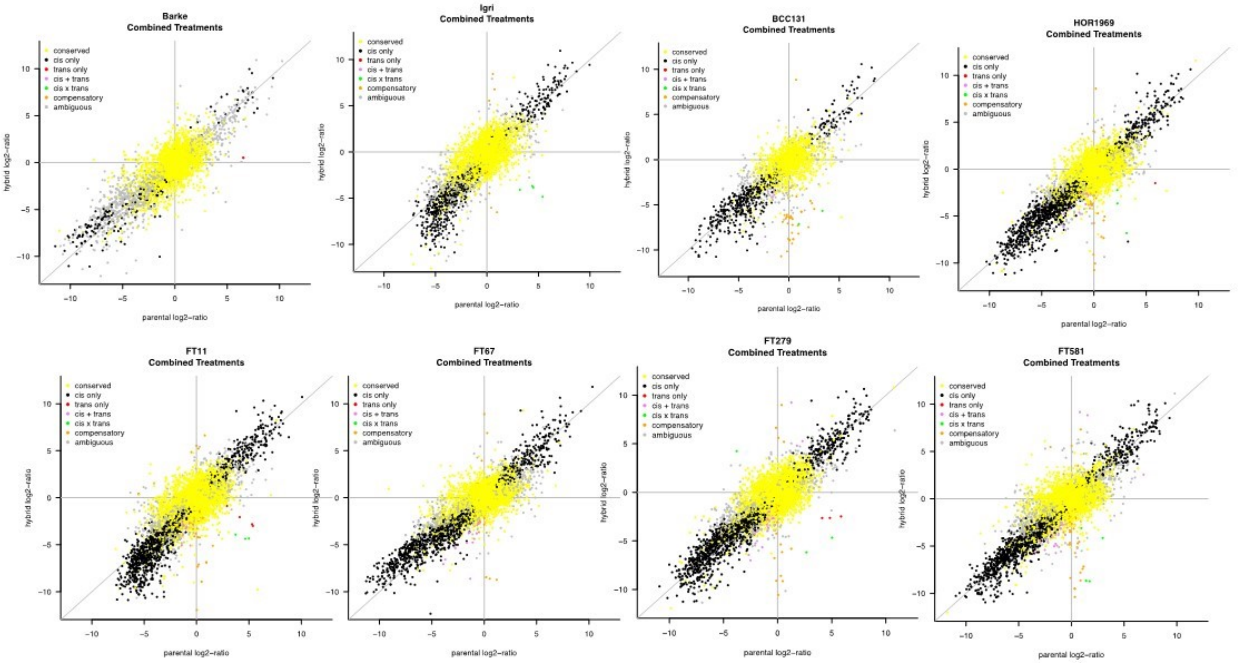

Figure S8. Log_2 ratio plots of parents (x-axis) vs. parental alleles in the hybrid (y-axis) for all crosses when treatments were not considered separately and instead grouped as additional replicates.

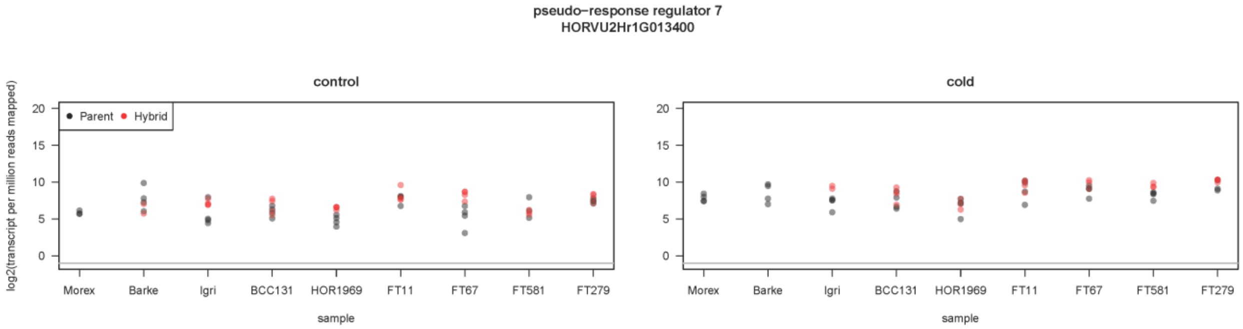

Figure S9. Expression (log_2-transformed transcripts per million reads mapped) values for parents (black) and hybrids (red) from each sample for *Ppd-H1* (HORVU2Hr1G013400). There is variation among the samples, but unlike the cold-responsive genes in Figure 5, there is no major difference between samples or treatments.

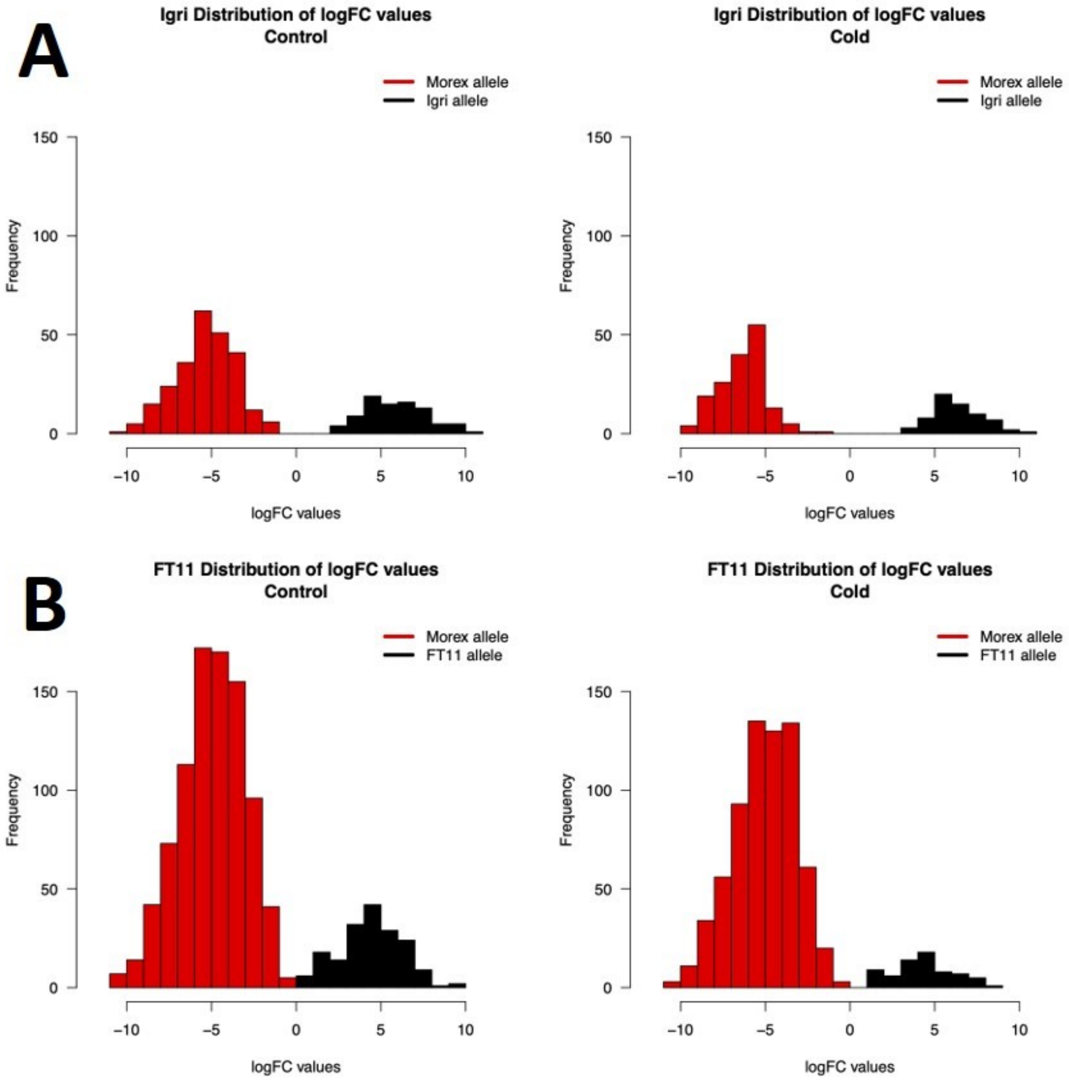

Figure S10. Distribution of $\log_2$ fold change values for two crosses, Morex × Igri and Morex × FT11. Log_2 fold change values for the maternal allele (Morex) are colored red while the values for the paternal allele (Igri or FT11) are colored black.
