# Supplementary material for: The contribution of cis- and trans-acting variants to gene regulation in wild and domesticated barley under cold stress and control conditions
Source: J Exp Bot. 2020 Jan 28;71(9):2573–84. doi: 10.1093/jxb/eraa036 (PMC7210754; doi:10.1093/jxb/eraa036)
Supplement: eraa036_suppl_Supplementary_Tables_S1-S9 [file eraa036_suppl_supplementary_tables_s1-s9.pdf]

Table S1. Accession numbers for individual accessions deposited into the European Nucleotide Archive (ENA). The table is sorted numerically according to the ENA accession number.

Type	Accession	Unique Name
Study	PRJEB29972	ena-STUDY-IPK-Gatersleben-27-11-2018-08:58:04:359-462
Sample	ERS2915967 (SAMEA5131586)	Sample_B_034
Sample	ERS2915968 (SAMEA5131587)	Sample_B_105
Sample	ERS2915969 (SAMEA5131588)	Sample_B_012
Sample	ERS2915970 (SAMEA5131589)	Sample_B_125
Sample	ERS2915971 (SAMEA5131590)	Sample_B_026
Sample	ERS2915972 (SAMEA5131591)	Sample_B_117
Sample	ERS2915973 (SAMEA5131592)	Sample_B_075
Sample	ERS2915974 (SAMEA5131593)	Sample_B_065
Sample	ERS2915975 (SAMEA5131594)	Sample_B_089
Sample	ERS2915976 (SAMEA5131595)	Sample_B_001
Sample	ERS2915977 (SAMEA5131596)	Sample_B_061
Sample	ERS2915978 (SAMEA5131597)	Sample_B_027
Sample	ERS2915979 (SAMEA5131598)	Sample_B_045
Sample	ERS2915980 (SAMEA5131599)	Sample_B_112
Sample	ERS2915981 (SAMEA5131600)	Sample_B_126
Sample	ERS2915982 (SAMEA5131601)	Sample_B_092
Sample	ERS2915983 (SAMEA5131602)	Sample_B_128
Sample	ERS2915984 (SAMEA5131603)	Sample_B_056
Sample	ERS2915985 (SAMEA5131604)	Sample_B_096
Sample	ERS2915986 (SAMEA5131605)	Sample_B_031
Sample	ERS2915987 (SAMEA5131606)	Sample_B_088
Sample	ERS2915988 (SAMEA5131607)	Sample_B_110
Sample	ERS2915989 (SAMEA5131608)	Sample_B_019

Sample	ERS2915990 (SAMEA5131609)	Sample_B_082
Sample	ERS2915991 (SAMEA5131610)	Sample_B_054
Sample	ERS2915992 (SAMEA5131611)	Sample_B_050
Sample	ERS2915993 (SAMEA5131612)	Sample_B_098
Sample	ERS2915994 (SAMEA5131613)	Sample_B_039
Sample	ERS2915995 (SAMEA5131614)	Sample_B_046
Sample	ERS2915996 (SAMEA5131615)	Sample_B_017
Sample	ERS2915997 (SAMEA5131616)	Sample_B_124
Sample	ERS2915998 (SAMEA5131617)	Sample_B_129
Sample	ERS2915999 (SAMEA5131618)	Sample_B_120
Sample	ERS2916000 (SAMEA5131619)	Sample_B_006
Sample	ERS2916001 (SAMEA5131620)	Sample_B_076
Sample	ERS2916002 (SAMEA5131621)	Sample_B_090
Sample	ERS2916003 (SAMEA5131622)	Sample_B_106
Sample	ERS2916004 (SAMEA5131623)	Sample_B_004
Sample	ERS2916005 (SAMEA5131624)	Sample_B_072
Sample	ERS2916006 (SAMEA5131625)	Sample_B_025
Sample	ERS2916007 (SAMEA5131626)	Sample_B_048
Sample	ERS2916008 (SAMEA5131627)	Sample_B_018
Sample	ERS2916009 (SAMEA5131628)	Sample_B_064
Sample	ERS2916010 (SAMEA5131629)	Sample_B_033
Sample	ERS2916011 (SAMEA5131630)	Sample_B_116
Sample	ERS2916012 (SAMEA5131631)	Sample_B_058
Sample	ERS2916013 (SAMEA5131632)	Sample_B_008
Sample	ERS2916014 (SAMEA5131633)	Sample_B_084
Sample	ERS2916015 (SAMEA5131634)	Sample_B_029

Sample	ERS2916016 (SAMEA5131635)	Sample_B_086
Sample	ERS2916017 (SAMEA5131636)	Sample_B_070
Sample	ERS2916018 (SAMEA5131637)	Sample_B_114
Sample	ERS2916019 (SAMEA5131638)	Sample_B_052
Sample	ERS2916020 (SAMEA5131639)	Sample_B_100
Sample	ERS2916021 (SAMEA5131640)	Sample_B_068
Sample	ERS2916022 (SAMEA5131641)	Sample_B_122
Sample	ERS2916023 (SAMEA5131642)	Sample_B_041
Sample	ERS2916024 (SAMEA5131643)	Sample_B_080
Sample	ERS2916025 (SAMEA5131644)	Sample_B_037
Sample	ERS2916026 (SAMEA5131645)	Sample_B_104
Sample	ERS2916027 (SAMEA5131646)	Sample_B_074
Sample	ERS2916028 (SAMEA5131647)	Sample_B_002
Sample	ERS2916029 (SAMEA5131648)	Sample_B_062
Sample	ERS2916030 (SAMEA5131649)	Sample_B_044
Sample	ERS2916031 (SAMEA5131650)	Sample_B_118
Sample	ERS2916032 (SAMEA5131651)	Sample_B_013
Sample	ERS2916033 (SAMEA5131652)	Sample_B_108
Sample	ERS2916034 (SAMEA5131653)	Sample_B_016
Sample	ERS2916035 (SAMEA5131654)	Sample_B_060
Sample	ERS2916036 (SAMEA5131655)	Sample_B_094
Sample	ERS2916037 (SAMEA5131656)	Sample_B_015
Sample	ERS2916038 (SAMEA5131657)	Sample_B_035
Sample	ERS2916039 (SAMEA5131658)	Sample_B_021
Sample	ERS2916040 (SAMEA5131659)	Sample_B_066
Sample	ERS2916041 (SAMEA5131660)	Sample_B_102

Sample	ERS2916042 (SAMEA5131661)	Sample_B_078
Sample	ERS2916043 (SAMEA5131667)	Sample_B_121
Sample	ERS2916044 (SAMEA5131668)	Sample_B_053
Sample	ERS2916045 (SAMEA5131669)	Sample_B_109
Sample	ERS2916046 (SAMEA5131670)	Sample_B_107
Sample	ERS2916047 (SAMEA5131671)	Sample_B_095
Sample	ERS2916048 (SAMEA5131672)	Sample_B_007
Sample	ERS2916049 (SAMEA5131673)	Sample_B_063
Sample	ERS2916050 (SAMEA5131674)	Sample_B_057
Sample	ERS2916051 (SAMEA5131675)	Sample_B_115
Sample	ERS2916052 (SAMEA5131676)	Sample_B_099
Sample	ERS2916053 (SAMEA5131677)	Sample_B_077
Sample	ERS2916054 (SAMEA5131678)	Sample_B_079
Sample	ERS2916055 (SAMEA5131679)	Sample_B_022
Sample	ERS2916056 (SAMEA5131680)	Sample_B_123
Sample	ERS2916057 (SAMEA5131681)	Sample_B_024
Sample	ERS2916058 (SAMEA5131682)	Sample_B_055
Sample	ERS2916059 (SAMEA5131683)	Sample_B_083
Sample	ERS2916060 (SAMEA5131684)	Sample_B_038
Sample	ERS2916061 (SAMEA5131685)	Sample_B_051
Sample	ERS2916062 (SAMEA5131686)	Sample_B_003
Sample	ERS2916063 (SAMEA5131687)	Sample_B_101
Sample	ERS2916064 (SAMEA5131688)	Sample_B_093
Sample	ERS2916065 (SAMEA5131689)	Sample_B_097
Sample	ERS2916066 (SAMEA5131690)	Sample_B_127
Sample	ERS2916067 (SAMEA5131691)	Sample_B_067

Sample	ERS2916068 (SAMEA5131692)	Sample_B_059
Sample	ERS2916069 (SAMEA5131693)	Sample_B_081
Sample	ERS2916070 (SAMEA5131694)	Sample_B_043
Sample	ERS2916071 (SAMEA5131695)	Sample_B_103
Sample	ERS2916072 (SAMEA5131696)	Sample_B_005
Sample	ERS2916073 (SAMEA5131697)	Sample_B_032
Sample	ERS2916074 (SAMEA5131698)	Sample_B_111
Sample	ERS2916075 (SAMEA5131699)	Sample_B_069
Sample	ERS2916076 (SAMEA5131700)	Sample_B_085
Sample	ERS2916077 (SAMEA5131701)	Sample_B_020
Sample	ERS2916078 (SAMEA5131702)	Sample_B_073
Sample	ERS2916079 (SAMEA5131703)	Sample_B_036
Sample	ERS2916080 (SAMEA5131704)	Sample_B_119
Sample	ERS2916081 (SAMEA5131705)	Sample_B_040
Sample	ERS2916082 (SAMEA5131706)	Sample_B_023
Sample	ERS2916083 (SAMEA5131707)	Sample_B_028
Sample	ERS2916084 (SAMEA5131708)	Sample_B_113
Sample	ERS2916085 (SAMEA5131709)	Sample_B_071
Sample	ERS2916086 (SAMEA5131710)	Sample_B_087
Sample	ERS2916087 (SAMEA5131711)	Sample_B_049
Sample	ERS2916088 (SAMEA5131712)	Sample_B_047
Sample	ERS2916089 (SAMEA5131713)	Sample_B_091
Sample	ERS2916090 (SAMEA5131714)	Sample_B_030
Sample	ERS2916091 (SAMEA5131715)	Sample_B_130
Sample	ERS2916092 (SAMEA5131716)	Sample_B_042
Sample	ERS2916093 (SAMEA5131717)	Sample_B_009

Table S2. Gene category assignment for Barke × Morex.

Control \ Cold	cis only	trans only	cis + trans	cis × trans	conserved	compensatory	ambiguous
cis only	8	0	0	0	2	0	273
trans only	0	0	0	0	0	0	0
cis + trans	0	0	0	0	0	0	0
cis × trans	0	0	0	0	0	0	0
conserved	0	0	0	0	3,738	0	231
compensatory	0	0	0	0	0	0	0
ambiguous	0	0	0	0	220	0	454

Table S3. Gene category assignment for Igri × Morex.

Control \ Cold	cis only	trans only	cis + trans	cis × trans	conserved	compensatory	ambiguous
cis only	172	0	0	0	34	0	134
trans only	0	0	0	0	0	0	0
cis + trans	0	0	0	0	0	0	0
cis × trans	0	0	0	1	0	0	0
conserved	18	0	0	0	3,713	0	193
compensatory	1	0	0	0	1	1	0
ambiguous	39	0	0	0	168	0	159

Table S4. Gene category assignment for BCC131 × Morex.

Control \ Cold	cis only	trans only	cis + trans	cis × trans	conserved	compensatory	ambiguous
cis only	289	0	0	0	23	1	55
trans only	0	0	0	0	3	0	0
cis + trans	1	0	1	0	0	1	0
cis × trans	0	0	0	1	1	1	0
conserved	21	0	0	0	1,771	3	111
compensatory	1	1	0	1	1	28	3
ambiguous	40	0	0	0	139	5	87

Table S5. Gene category assignment for HOR1969 × Morex.

Control \ Cold	cis only	trans only	cis + trans	cis × trans	conserved	compensatory	ambiguous
cis only	617	0	1	0	55	2	136
trans only	0	1	0	0	1	0	1
cis + trans	7	0	7	0	0	1	0
cis × trans	0	0	0	0	0	1	0
conserved	62	0	0	0	5,165	1	200
compensatory	0	0	1	0	1	17	3
ambiguous	96	0	0	0	292	3	179

Table S6. Gene category assignment for FT11 × Morex.

Control \ Cold	cis only	trans only	cis + trans	cis × trans	conserved	compensatory	ambiguous
cis only	641	1	4	0	191	2	226
trans only	0	0	0	0	0	0	1
cis + trans	1	0	3	0	0	2	1
cis × trans	0	1	0	1	2	1	0
conserved	35	1	0	0	7,047	1	194
compensatory	0	0	0	0	1	20	8
ambiguous	71	0	0	0	630	3	229

Table S7. Gene category assignment for FT67 × Morex.

Control \ Cold	cis only	trans only	cis + trans	cis × trans	conserved	compensatory	ambiguous
cis only	754	0	5	0	51	1	151
trans only	1	0	0	0	2	0	0
cis + trans	3	0	9	0	0	2	0
cis × trans	0	0	0	0	0	0	0
conserved	41	0	0	0	6,408	0	255
compensatory	0	0	0	0	0	12	5
ambiguous	105	0	0	0	484	1	300

Table S8. Gene category assignment for FT279 × Morex.

Control \ Cold	cis only	trans only	cis + trans	cis × trans	conserved	compensatory	ambiguous
cis only	749	0	5	0	45	2	93
trans only	0	0	0	1	1	0	0
cis + trans	3	0	12	0	0	0	0
cis × trans	0	0	0	0	1	2	0
conserved	78	0	0	0	6,241	2	289
compensatory	0	0	1	0	2	19	6
ambiguous	137	1	0	1	365	4	222

Table S9. Gene category assignment for FT581 × Morex.

Control \ Cold	cis only	trans only	cis + trans	cis × trans	conserved	compensatory	ambiguous
cis only	789	0	9	0	43	0	111
trans only	2	0	0	0	1	0	1
cis + trans	4	0	6	0	0	2	0
cis × trans	0	0	0	2	1	4	2
conserved	81	1	0	0	6,582	0	360
compensatory	0	0	3	1	1	20	4
ambiguous	157	0	0	1	465	3	283
